# Reward Deficiency Syndrome (RDS): A Cytoarchitectural Common Neurobiological Trait of All Addictions

**DOI:** 10.3390/ijerph182111529

**Published:** 2021-11-02

**Authors:** Kenneth Blum, Abdalla Bowirrat, Eric R. Braverman, David Baron, Jean Lud Cadet, Shan Kazmi, Igor Elman, Panyotis K. Thanos, Rajendra D. Badgaiyan, William B. Downs, Debasis Bagchi, Luis Llanos-Gomez, Mark S. Gold

**Affiliations:** 1 Division of Addiction Research & Education, Center for Psychiatry, Medicine, & Primary Care (Office of the Provost), Western University Health Sciences, Pomona, CA 91766, USA; dbaron@westernu.edu (D.B.); shan.kazmi@westernu.edu (S.K.); 2 Institute of Psychology, Eotvos Loránd University, 1053 Budapest, Hungary; 3 Division of Nutrigenomics, Synaptamine Inc., Austin, TX 78701, USA; 4 Department of Psychiatry, Wright State University Boonshoft School of Medicine and Dayton VA Medical Center, Dayton, OH 45435, USA; 5 Department of Psychiatry, University of Vermont, Burlington, VT 05405, USA; 6 Division of Precision Addiction Management, Geneus Health (Division of Ivitalize Inc.), San Antonio, TX 78249, USA; 7 Division of Nutrigenomics, The Kenneth Blum Behavioral & Neurogenetic Institute (Division of Ivitalize Inc.), Austin, TX 78701, USA; pathmedical@gmail.com (E.R.B.); billd@vni.life (W.B.D.); debasisbagchi@gmail.com (D.B.); luisllanos522@gmail.com (L.L.-G.); 8 Department of Molecular Biology, Adelson School of Medicine, Ariel University, Ariel 40700, Israel; bowirrat@gmail.com; 9 Molecular Neuropsychiatry Research Branch, NIH National Institute on Drug Abuse, Bethesda, MD 20892, USA; jcadet@intra.nida.nih.gov; 10 Department of Psychiatry, Harvard School of Medicine, Cambridge, MA 02115, USA; dr.igorelman@gmail.com; 11 Behavioral Neuropharmacology and Neuroimaging Laboratory on Addictions, Clinical Research Institute on Addictions, Department of Pharmacology and Toxicology, Jacobs School of Medicine and Biosciences, State University of New York at Buffalo, Buffalo, NY 14260, USA; thanos@buffalo.edu; 12 Department of Psychiatry, South Texas Veteran Health Care System, Audie L. Murphy Memorial VA Hospital, San Antonio, TX 78229, USA; badgaiyan@gmail.com; 13 Long School of Medicine, University of Texas Medical Center, San Antonio, TX 78229, USA; 14 Department of Pharmaceutical Sciences, College of Pharmacy, Southern University, Houston, TX 77004, USA; 15 Department of Psychiatry, Tulane University School of Medicine, New Orleans, LA 70118, USA; drmarksgold@gmail.com; 16 Department of Psychiatry, Washington University School of Medicine, St. Louis, MO 63130, USA

**Keywords:** dopamine homeostasis, precision addiction management, GARS, KB220, reward deficiency syndrome (RDS), neuroimaging

## Abstract

Alcohol and other substance use disorders share comorbidity with other RDS disorders, i.e., a reduction in dopamine signaling within the reward pathway. RDS is a term that connects addictive, obsessive, compulsive, and impulsive behavioral disorders. An estimated 2 million individuals in the United States have opioid use disorder related to prescription opioids. It is estimated that the overall cost of the illegal and legally prescribed opioid crisis exceeds one trillion dollars. Opioid Replacement Therapy is the most common treatment for addictions and other RDS disorders. Even after repeated relapses, patients are repeatedly prescribed the same opioid replacement treatments. A recent JAMA report indicates that non-opioid treatments fare better than chronic opioid treatments. Research demonstrates that over 50 percent of all suicides are related to alcohol or other drug use. In addition to effective fellowship programs and spirituality acceptance, nutrigenomic therapies (e.g., KB220Z) optimize gene expression, rebalance neurotransmitters, and restore neurotransmitter functional connectivity. KB220Z was shown to increase functional connectivity across specific brain regions involved in dopaminergic function. KB220/Z significantly reduces RDS behavioral disorders and relapse in human DUI offenders. Taking a Genetic Addiction Risk Severity (GARS) test combined with a the KB220Z semi-customized nutrigenomic supplement effectively restores dopamine homeostasis (WC 199).

## 1. Introduction

Reward Deficiency Syndrome (RDS) is a term that connects addictive, compulsive, and impulsive behavioral disorders. This group of behaviors has been associated with genetic variants and epigenetic (environmental) changes that result in an inadequacy in the neurotransmission of reward or pleasure. RDS as a concept was developed through extensive animal and human research that sought to link genetic, environmental and behavioral patterns through the field of behavioral genetics [1]. Such research paved the way for groundbreaking discoveries that shed light upon the molecular biology of the underlying neurotransmission alcoholism and indeed, all addictive and compulsive behaviors. Understanding this concept-elucidated in the forthcoming sections is central to treating substance and behavioral addictions. The RDS phenomena has wide-ranging implications in not only addictive and compulsive behavior treatments but also in the relevance of “Free-Will as a functional empirical concept in perennial discussion of philosophical ethics.

An ongoing controversy concerning addiction is if it is guided by moral choice (free will). Do addicts engage in drug use in the absence of genetic antecedents, or is the opposite true? Drug addiction might be a consequence of a pre-existing propensity or vulnerability. However, a causative agent may also induce a vulnerability [2]. Thus, the proponents of controlled drinking would have to agree that drug abuse can induce epigenetic insults that impact normal reward processing, further affecting the propensity to addiction. Generally, these same proponents do not call for controlled methamphetamine administration as it is clear that any degree of use can be neurotoxic [3]. These known insults to the brain cause alterations that make abstinence less likely and lead to the escalation of increased drug usage in the face of withdrawal symptomatology and tolerance. The psychiatry, neurology, and neuroscience communities agree that epigenetic insults, both positive (deacetylation) or negative (methylation), induces an unwanted state affecting gene expression (up or down) due to mRNA transcription errors targeted to specific reward genes [4]. New evidence may emerge from locus-specific neuro-epigenetic editing—which is an innovative method for determining the causal epigenetic molecular mechanisms that drive a state of addiction. Further understanding in this area will increase the field’s ability to uncover the precise epigenetic mechanisms underlying drug addiction. This may lead to novel treatments for addictive disorders. 

To help comprehend the evidence in favor of a trait (genetic) basis for drug and non-drug behavioral addictions, we provide herein a brief description of the legal concepts related to the terms “*determinism*” and *free will*”. In today’s Judicial System, our legal apparatus operates according to an “as if” theory. This approach accepts the truth of determinism yet adopts an “as if” view of human freedom. In other words, society should design institutions “as if” human action was not determined. Proponents of this scheme recognize that although determinism may be the first postulate of science, to choose free action as the first postulate of legal and moral thought challenges the current philosophy of ignoring predispositions based on, for example, Reward-Gene variations (polymorphisms). Along these lines, Tikkanen et al. [5] offer a thought-provoking report whereby they found that carriers of the MAOA-H (high activity) allele have a high risk for committing severe, recidivistic, impulsive, violent crimes after exposure to heavy drinking and Childhood Physical Abuse (CPA). In essence, under the “as if” theory, the legal system attempts to reconcile the two paradigms by working out a form of “rough justice”. The system presumes free will and imputes criminal responsibility but also allows for the uncontrollable influence of determinism by providing exculpatory defenses or by mitigating resulting punishment. As neuroscientists, geneticists, and clinicians, it is this latter concept that we consider the area of change that requires deep thinking in the absence of legal precedent. Courts have responded with varying degrees of receptivity to scientific evidence suggesting a causal link between human behavior and predetermined biological factors. A genetic defense claim implies impairment of free will; however, the judicial system has been relatively consistent in alignment with defenses based on insanity and diminished mental capacity. It is challenging and rare to successfully use an “insanity” or “diminished capacity” defense in many states. For example, a study conducted in multiple states reported that insanity defense claims were raised in one percent of felony cases, of which only 26% resulted in acquittal [6]. Moreover, Utah, Montana, Idaho, and Kansas have abolished the insanity defense outright [7]. The logic behind the insanity defense is obvious; put simply, “the insanity defense accepts that most people act under Free Will but allows leeway for a person who is incapable of making decisions based on acceptable moral and legal standards. 

However, our group has found precedential evidence in criminal court to assist the courts in choosing rehabilitation vs. incarceration for DWI recidivism; specifically, probation, treatment for substance use disorder (SUD), and continued monitoring of treatment (rehabilitation) as an alternative to other sentencing. The determining factors for eligibility for alternative sentencing are based substantially on the Genetic Addiction Risk Severity (GARS) test. This is the first case study to objectively point to genetically based ‘determinism’ rather than ‘free will’ in determining sentencing for a DWI offender [8]. 

Historically, the original concept of alcoholism as a possible disease dates back to the early thirties as espoused by Bill Wilson in the Big Book and Jellinek in 1960 [9]. In brief, Jellinek viewed alcoholism as an epigenetic phenomenon (not a genetically induced trait). Jelinek concluded that there were five types of alcoholism: Alpha—psychological dependence; Beta-medical consequences including liver and nerve disorders; Gamma-increase tissue tolerance; Delta-inability to abstain; Epsilon-binge drinking. However, Jelinek’s theory left three basic questions unanswered:

1. What are the underlying biogenetic mechanisms that cause alcoholism?
2. Do stress and social influences alter cell function and lead to alcoholism?
3. Does long-term excessive drinking alter cell function and lead to alcoholism?

In essence, Jelinek [10] made it clear that epigenetics played a role in alcoholism and paved the way for extensive molecular neurobiological experiments to help answer these complicated questions. 

### 1.1. Alpha—Psychological Dependence

There are, of course, those who disagree. Indeed, a replay of an old controversy about the underlying nature of alcoholism is now underway, and the debate continues [11]. On one side are the scientists whose research is in the field of Psychiatric Genetics. The first evidence for a genetic connection to alcoholism by Blum and Noble and associates involved the dopamine D2 receptor (***DRD2***) ***Taq 1*** allele and severe alcoholism in 1990 [12], boasting 24,890 articles listed in PUBMED (1/3/21). This enormous amount of work (over five decades) points to a biogenetic disease characterized by genetic anomalies, leading to biochemical deficiencies or imbalances and receptor malfunctions. On the other side are a number of psychologists who seem to ignore a vast body of research findings spanning the past five decades [13,14,15,16]. They reject or are unaware of important clinical data, the concept of SUDs as a disease, and in our opinion, have advanced three misleading hypotheses.

**Hypothesis** **1.** *Total abstinence from alcohol and other psychoactive drugs is not necessary for recovery*.

This hypothesis seems to have been derived, in part, from an early experiment in 1962 by D. L. Davies, who studied “normal drinking in recovered alcohol addicts”. His findings were cited in the American Psychologist in 1983 by Alan Marlatt at the University of Seattle. Davies sent shock waves through the alcoholism field by publishing the results of a long-term follow-up of patients treated for alcoholism at the Maudsley Hospital in London. Davies [17] challenged the traditional emphasis on total abstinence as the only viable “cure” for alcoholism by showing that of 93 male alcoholics who were followed up for a period of 7 to 11 years after treatment, 7 reported a pattern of normal drinking. One problem with Marlatt’s interpretation of this study is that it ignores the stated fact that seven out of 93 subjects is less than 8 percent, a tiny fraction. Furthermore, he seems to have overlooked Davies’ own final conclusion at the end of his report: “It is suggested that such cases are more common than has hitherto been recognized and that the generally accepted view that no alcohol addict can ever again drink normally should be modified, although all patients should be advised to aim at total abstinence”.

The Betty Ford Institute convened a panel of famous experts to consider what recovery was and how abstinence was related and integral to recovery (2009). We know from a recent Stanford metanalysis that A.A. works, abstinence is a central and integral part of recovery and that recovery can be sustained using A.A. and similar approaches [18]. The most important fallacy behind the data cited by Marlatt was revealed in 1985 when Griffith Edwards conducted a follow-up three decades later on the seven subjects in Davies’ sample (who were supposed to have been able to drink normally). Edwards found that of the seven alcoholic men, five had resumed destructive drinking patterns; three of the five had also used psychoactive drugs heavily; three of the five had been using alcohol abnormally even during Davies’ study. One of the seven eventually experienced Wernicke–Korsakoff syndrome, a form of alcohol-related brain damage; one was hospitalized for peptic ulcers; one experienced liver enlargement due to heavy drinking [19]. The data in support of controlled drinking have a way of disintegrating when they are examined closely.

**Hypothesis** **2.** *Alcoholism is not a disease but merely a pattern of learned behavior*.

This hypothesis was derived mainly from the much-publicized work of Mark and Linda Sobell at Patton State Hospital in the California Department of Mental Hygiene in Patton, California. They attempted to prove that alcoholics could be *taught* controlled drinking skills and that these skills would be effective outside the hospital environment. Again, the underlying thesis was that abstinence is not essential to recovery. The Sobell’s reported on a group of 20 alcoholic patients who had received behavioral therapy aimed at moderating their drinking patterns. In a follow-up study lasting two years, they claimed that 19 patients were successfully practicing controlled drinking [20]. One of the leading proponents of controlled drinking is Herbert H. Fingarette in the Department of Philosophy at the University of California in Santa Barbara. In his book *Heavy Drinking: The Myth of Alcoholism as a Disease,* he referred to the Sobell’s’ work in the following words, “In 1973, Mark Sobell and Linda Sobell issued their groundbreaking report detailing the successful result of their elaborate and carefully evaluated program of controlled drinking”. Summing up his own views on the subject, he said, “Controlled drinking has become the umbrella term for the notion that abstinence may not be the only reasonable goal for the heavy drinker seeking help”. [21]. Unfortunately for the proponents of controlled drinking, the Sobell’s’ work did not stand up to closer scrutiny. On 9 July 1982, *Science* published a ten-year follow-up of the Sobell’s’ evidence by Pendery et al. [22] in the Department of Psychiatry at UCLA. The study showed that 13 of the 20 Sobell subjects were hospitalized again within a year, and three others had used alcohol destructively during the study period. The other four subjects were found to be psychologically (not physically), dependent on alcohol. Of these four, three had a record of repetitive arrests on drunk charges. Only one of them seemed capable of indulging in controlled drinking, and this individual may have been misdiagnosed as an alcoholic. 

However, a more recent article by the Sobell’s provided some new evidence to suggest that some “alcoholics” can acquire and maintain controlled drinking behaviors. Even with the caveat of the word “some”, they continue to imply that traditional treatment of alcoholics may be handicapped by unvalidated beliefs concerning the nature of the disorder [23].

**Hypothesis** **3.** *Alcoholism can be arrested and often cured*.

To test this hypothesis, among others, the Rand Corporation, under contract from NIAAA, carried out two studies of alcoholism (one in 1976 and the other in 1981). The first report evaluated 597 alcoholics for 18 months after they had completed treatment. The authors found that 24% were abstaining, and 22% were drinking normally [24]. This report was greeted with pleasure by adherents of the controlled drinking doctrine. For example, Morris Chafetz, former director of the NIAAA stated: “The Rand Report should make those interested in the plight of alcoholic people jump for joy” [25] Samuel B. Guze, head of the Department of Psychiatry at the Washington University School of Medicine, said: “What the data demonstrate is that remission is possible in many alcoholics and that many of these are able to drink normally for an extended period”; however, the research methodology and conclusions of the first Rand Report were considered highly suspect by major scientists in the field. 

Ernest Noble, at that time director of the NIAAA, was particularly concerned about the treatment community: “Until further definite scientific evidence exists to the contrary, I feel that abstinence must continue as the appropriate goal in the treatment of alcoholism. Furthermore, it would be extremely unwise for a recovered alcoholic to even try to experiment with controlled drinking” [26].

Others at that time also commented on the report—“The first Rand Report was so methodologically inadequate that nothing could be concluded from it. The Report was seriously marred by an enormous loss to follow-up rate, sample, bias on outcome, unreliable and invalid-measurements of quantity and frequency of consumption, loss of entire treatment centers from the original sample of centers, shoddy data-gathering procedures by treatment staff and a follow-up window of such short duration (30 days of drinking behavior) that it was an embarrassment [27]. When the Second Rand Report was released, it claimed that nearly 40 percent of the subjects were drinking normally after four years. Like those in the First Report, these results appeared to indicate that alcoholism can, indeed, be arrested and perhaps cured. But the Second Report came under academic attack as well, for example by John Wallace again:

The Second Report was methodologically superior to the [First Report]. However, when the results are examined for sustained non-problem drinking over time and are corrected for invalid measurement of quantity/frequency [of drinking], the best estimate of the sustained non-problem drinking rate is around 3 percent to 4 percent. In short, at least 96 percent of the Second Rand Report subjects failed to give evidence of sustained non-problem drinking over the four-year follow-up period. This is hardly an advertisement for the success of controlled drinking in alcoholics.

In their efforts to discredit the disease concept, the proponents of controlled drinking resorted to strange claims. For example, Stanton Peele (who suggested that the Blum & Noble study in JAMA (1990) was fraudulent and presented no evidence in the process) wrote in *The Sciences*, stating that the disease concept fosters irresponsibility and provides an excuse for continued drinking. Peele also claimed that people are placed into treatment centers merely for getting drunk a few times after years of moderate drinking [28]. Anyone familiar with the modem treatment centers would be excusably disturbed by this statement. John Wallace summed it up succinctly—“the disease model-including genetic, neurochemical, and cultural factors are taught to patients to help them understand their illness”. John Wallace also pointed out that among the 12,000 patients treated in his program, “responsibility” is emphasized during treatment. Upon graduation, a medallion is awarded to each patient and inscribed [29]. With modern third-party payment and stringent criteria, there is no chance that a person would be admitted for treatment in the absence of a significant prior history of alcohol or drug abuse. 

### 1.2. Beta-Medical Consequences including Liver and Nerve Disorders

Roughly 3.3 million deaths (5.9% of all deaths) are believed to be due to alcohol each year [30]. Alcoholism, which is defined as the recurring, deleterious use of alcohol despite its negative consequences, has a lifetime prevalence of 17.8% [31]. Alcoholism is generally any drinking of alcohol that results in significant mental or physical health problems [32]. Excessive alcohol usage can damage all organ systems, mainly affecting the liver, heart, pancreas, and brain. Further health consequences include stroke, high blood pressure, atrial fibrillation, sexual dysfunction, malabsorption, and deleterious effects to the immune system (Diagnostic and statistical manual of mental disorders: DSM-5 [33]. One potential reason for reduced immune response may be that excessive alcohol intake reduces the synthesis of endorphins/enkephalins with a resultant decrease in immunity [34]. We know this from experiments conducted with Golden Syrian hamsters; each animal was placed in a cage with three drinking bottles: one containing water, one containing water and ethanol, and one empty. Control hamsters received water only. After a year, the experimental hamsters showed a significantly lower concentration of a leucine-enkephalin-like immunoreactive substance in the basal ganglia than the control hamsters. This finding indicates that ethanol’s long-term (approximately 20 years in humans) action involves endogenous peptidyl opiates.

Moreover, alcoholism can result in mental illness, delirium tremens, Wernicke –Korsakoff syndrome, irregular heartbeat, polyneuropathy, liver cirrhosis, and enhanced cancer risk [35]. Drinking during pregnancy can lead to fetal alcohol spectrum disorders [36]. Generally, women are more sensitive than men to the harmful effects of alcohol, primarily due to their higher proportion of body fat, smaller body weight, and lower capacity to metabolize alcohol. In comparison to men, more women are lifetime abstainers, drink less, and are less likely to engage in problem drinking, develop alcohol-related disorders or alcohol withdrawal symptoms. However, women who drink excessively develop more medical problems. Biological (sex-related) factors, including differences in alcohol pharmacokinetics and its effect on brain function and the levels of sex hormones, may contribute to some of those differences. In addition, differences in alcohol effects on behavior may also be driven by psycho-socio-cultural (gender-related) factors [37]. For a small number of individuals, prolonged, severe alcohol abuse ultimately leads to dementia [38].

Moreover, social skills can be significantly impaired in people with alcoholism due to the neurotoxic effects of alcohol on the brain, particularly on the prefrontal cortex area of the brain [39]. In fact, Murano et al. [31] discovered “pseudo-immature” brain cell states of the dentate gyrus and prefrontal cortex (PFC) in mouse models of epileptic seizure and psychotic disorders. Similar pseudo-immaturity has been recognized in patients with bipolar disorder and schizophrenia. Patients with alcoholism occasionally exhibit similar psychological symptoms, implying shared molecular and cellular mechanisms between these illnesses [40]. 

### 1.3. Gamma-Increase Tissue Tolerance

Escalating doses of alcohol occur during chronic alcohol abuse, and there is evidence that genetic antecedents may play a role in its development in humans. However, it is indeed interesting that “genotype” related to, for example, the rodent endophenotype of alcohol-preferring may be linked to innate tolerance. Elston et al. [41] compared three strains of mice (ICR Swiss, DBA/2J, and C57Bl/6J) for initial sensitivity, recovery from intoxication, and acute tolerance development to ethanol. The C57Bl/6J mice (high alcohol-preferring) were less sensitive and recovered more rapidly from the effects of the same dose of ethanol than the other two strains treated. This similar finding was confirmed in humans by Schuckit et al. [42]. Their study involved measuring body sway or static ataxia in 34 drinking but non-alcoholic men (age 21–25) who have an alcoholic first-degree relative (the family-history-positive, FHP, or negative family history -FHN). Post-acute alcohol intake revealed that the increase in body sway was significantly less for the FHP than for the FHN group. The authors concluded that their results “*are consistent with the significantly less intense subjective feelings of intoxication after drinking for the FHP men, and also parallel findings of less intense ethanol-related changes in biologic and cognitive test scores*”. Importantly, similar to the Elston et al. [41] study, in rodents, a decreased intensity of the [reaction to ethanol should be explored further as a possible genetic trait marker of a predisposition toward alcoholism and argues in favor of genetic antecedents to cellular tolerance induced by chronic alcohol intake.

In terms of cellular mechanisms related to the acute effects, compared to its chronic effects, we refer to the work of Diamond et al. [43]. It is well known that exposure to ethanol in culture inhibits adenosine uptake into cells, thereby increasing extracellular adenosine concentration. The extracellular adenosine then reacts with adenosine A2 receptors to stimulate intracellular cAMP production. However, during prolonged exposure to ethanol, the increase in cAMP is followed by the development of heterologous desensitization (tolerance) of receptors coupled to adenylyl cyclase through Gs, the stimulatory GTP-binding protein. Ethanol-induced heterologous desensitization seems to be due to a reduction in mRNA and protein for G alpha s, a subunit of Gs. Diamond et al. [43] also point out that there appears to be a synergism between ethanol-induced heterologous desensitization of receptor-stimulated cAMP production (cellular dependence) and resistance to ethanol inhibition of adenosine uptake (cellular tolerance). This is because both lead to reduced intracellular levels of cAMP.

Moreover, adenosine receptor-dependent cAMP production regulation may be altered in patients at risk of developing alcoholism because of genetic factors [43]. Along these lines, Hack & Christie [44] suggested that, unlike opiates and cocaine, adenosine receptor activation worsens the behavioral effects of drug intake. There is also evidence that demonstrates that substances that negatively modulate adenosine receptor function have some utility in attenuating the impact of ethanol use. Finally, in terms of genetic antecedents and ethanol-induced tolerance, lymphocytes from alcoholics cultured from many generations, in the absence of ethanol, show increased adenosine receptor-dependent cAMP production and increased sensitivity to ethanol-induced heterologous desensitization [45]. 

### 1.4. Delta-Inability to Abstain

Some suggest that drug abuse and alcoholism are not diseases at all and that they are not consequences of a brain disorder, as expressed recently by the American Society of Addiction Medicine (ASAM). One can argue that addicts can abstain on their own and moderate their drug and alcohol intake. When addicts present to a treatment program or enter the 12 Step Program & Fellowship, many can finally achieve complete abstinence. Through screening research and surveys, the consensus suggests that alcoholism reduces a person’s life expectancy by around ten years [46]. A high rate of suicide is observed in chronic alcoholics, which increases the longer a person abuses alcohol. Approximately 3–15 % of alcoholics commit suicide [47], and research has determined that over 50 percent of all suicides are related to alcohol or other drug use. This is thought to be due to alcohol causing physiological distortions of neurochemistry, as well as social isolation. Suicide is also widespread in adolescent alcohol abusers, with 25 percent of suicides in adolescents being related to alcohol abuse [47,48]. The results of a long-term (60-year) follow-up study of alcoholic men revealed a “return to controlled drinking rarely persisted for much more than a decade without relapse or evolution into abstinence.” [49]. Accordingly, the idea that there was also a “return-to-controlled drinking, as reported in short-term studies, is often a mirage” [49].

A summary of Valliant’s findings regarding A.A. is presented herein:

- A.A. is a good fit for a small number of people with alcohol problems and helps them to abstain.
- A.A. is a poor fit for the majority of people with alcohol problems and can make some people worse.
- A.A. is better at creating “true believers” than it is at eliminating problem drinking.
- Whether or not A.A. is a good fit for a person has little to do with the frequency a person drinks or the number of alcohol-related problems that a person has. Importantly, they have the right personality type.
- A.A. is a good fit for black-and-white thinkers who accept proof by authority.
- A.A. is a poor fit for people who think in shades of gray and demand experimental evidence and scientific proof.

Since Valliant has assessed the potential of the 12 steps in reducing relapse, there have been many reports to the contrary demonstrating the importance of meditation, transcendence, mindfulness, spirituality, and personality. Our group revealed a direct relationship between remission to substance abuse and belief in spirituality [50]. Some studies have been directed at understanding spirituality from a neurotransmitter level. Finnish scientists discovered no association between 5-HT-1A receptors and spiritual experiences in both patients with major depression and healthy controls [51], while others did find an association. Specifically, Borg et al. [52] revealed that spiritual acceptance has a significant correlation with the density of 5-HT-1A receptors and “may explain why people vary greatly in spiritual zeal”. On a similar note, boys and girls with the combination of the short 5-HTTLPR, and homozygosity for the long AP-2beta genotype had significantly reduced scores on Self-Transcendence and Spiritual Acceptance [53]. Other work by Davidson’s group on mindfulness demonstrates the impact of meditation on the brain’s reward circuitry. They determined that expert meditators activated the right medial frontal, middle temporal, precentral and postcentral gyri and the lentiform nucleus to a greater degree on the fMRI adapted Stroop Word-Color Task (SWCT), which assesses attention and impulse control. This could suggest that meditation coupled with enhanced spiritual belief may generate DA release at the cingulate gyrus and VTA that could translate to better clinical outcomes and reduced relapse [54,55]. 

However, if controlled drinking fails, then there may be successful alternatives that fit particular individuals. Unfortunately, those with known genetic antecedents for vulnerability to substance abuse require abstinence [56]. In previous publications from our group, we have attempted to establish personal differences in recovery by determining the molecular neurobiological basis of each step of the 12 Step program. Blum et al. [57] explored how the molecular neurobiological basis of the 12 Step program can impact alcoholism and other addictive behaviors, despite the presence of addiction risk gene polymorphisms. This exploration has already been completed in part by Blum and others in a 2013 Springer Neuroscience Brief. Briefly, the neurobiological, genetic, and molecular links to epigenetic changes in individuals who regularly attend A.A. meetings have heuristic value. It begs the question in terms of whether “12 Step programs and Fellowship” bring about neuroplasticity and continued dopamine D2 receptor proliferation, despite having hypodopaminergic type polymorphisms such as the DRD2 A1 allele. “Like-minded” doctors of ASAM are aware that patients in treatment, without the “psycho-social-spiritual trio”, may not be acquiring the critical benefits maintained by adopting 12 Step doctrines. Do we benefit from coupling medication-assisted treatment (MAT) that favors combining dopamine agonist modalities (DAM) as possible histone-deacetylase activators with the 12 Steps, followed by a program that embraces either one or the other?

### 1.5. Epsilon-Binge Drinking

Excessive alcohol intake or binge drinking is an unwanted serious, preventable public health problem in the United States and globally. Alcohol and other substance use disorders share comorbidity with more generalized reward deficiency disorders, characterized by a reduction in dopamine signaling within the reward pathway. One way to evaluate the role of compounds on binge drinking is to employ the darkroom test first identified in the 70s [58,59,60,61]. Along these lines, it has now been determined that two novel key molecules-**kcnk_13_ and rasgrf_2_** have essential functions related to ethanol binge drinking in animal models and even young adults. Based on You et al. [62], we hypothesize that identifying risk polymorphisms of kcnk_13_ and rasgrf_2_ coupled with genetic addiction risk score (GARS) guided precision pro-dopamine regulation will mitigate binge ethanol drinking. We previously published reports on the benefits of this unique approach and provided outcome data in both binge-drinking animals and human DUI offenders, showing reductions in alcohol intake and relapse prevention [63]. Furthermore, since binge drinking has been associated with traffic accidents, leading to incarceration instead of potential rehabilitation, there is preliminary evidence to suggest that utilization of GARS in terms of defense, whereby “determinism” overrides “free will”, seems plausible [8]. 

A review of the literature revealed that both **kcnk_13_ and rasgrf_2_** ion channels, which may modulate dopaminergic function, are involved in regulating unwanted binge drinking [62,64]. In the case of a **rasgrf_2_** haplotype containing rs26907, this SNP is associated with a decreased reward sensitivity and number of binge drinking episodes in adolescent boys. However, after a careful search of the literature to our knowledge, we could not find any reference to a **kcnk_13_** polymorphism. Moreover, based on previously published data, there is ample evidence that KB220 variant significantly attenuates binge drinking in P rodents [63] and significantly reduces relapse in human DUI offenders to alcohol (only 17 percent relapsed in 10 months) [65]. In other work, it was also shown that a DUI offender female with subsequent testing of GARS coupled with a semi-customized precision KB220 variant resulted in a significant positive clinical outcome [66]. In addition, KB220Z displays increased functional connectivity and volume across specific brain regions involved in dopaminergic function across the brain circuitry in naïve rodents [67]. Finally, it is particularly noteworthy that ongoing research related to the question of utilizing “Precision Addiction/Behavioral Management” to dissect the age-old legal question of “determinism” vs “free -will” [8] is now being addressed in drug court from research in our laboratory [68]. 

**What are the underlying biogenetic mechanisms that cause addictive behaviors?**

***Background and stats:*** ***The disease model as overlapping addictions from birth to adulthood.***

It is noteworthy that changes in the nomenclature of addictions mandate a considerable shift in the conceptualization of addictions, including non-substance-related behaviors. A significant amount of data indicates that there are overlaps of different types of addictive behaviors in phenomenology, etiology, and the underlying psychological and biological mechanisms. Along these lines, Kotyuk et al. [40] analyzed data from 3003 adolescents and young adults (42.6% males; mean age 21 years). Associations were identified between (I) smoking and problematic Internet use, eating disorders, gambling, and exercising (II) alcohol consumption and problematic Internet use, online gaming, gambling, and eating disorders, and (III) cannabis use and problematic online gaming and gambling. The results indicate a considerable overlap between the occurrence of these addictions and behaviors. The results highlight the importance of investigating the possible common genetic, psychological, and neural pathways. These data further support concepts such as the Reward Deficiency Syndrome and the component model of addictions that propose a common phenomenological and etiological background of different addictive and related behaviors.

It is essential to realize that while we are cognizant that all addictive behaviors (drug and non-drug) are globally widespread and a major problem for most countries, we are compelled to highlight the worst opioid crisis ever in America with a message of hope. We must also point out that there is an increase in psychostimulant and benzodiazepine dependence and, as always, alcoholism [68,69,70]. Pain is an extensive public health problem that costs society at least 560–635 billion dollars annually, which is an amount equal to about 2000 dollars for every person living in the United States. This would include the total incremental cost of health care due to lost productivity from pain (based on hours of work lost, days of work missed, and lower wages), which ranges between 261 to 300 billion dollars and 297–336 billion dollars. The solution has been to prescribe opioid medications to reduce pain. However, the United States is in the midst of an opioid overdose epidemic in the face of the COVID 19 pandemic. From 1999 to 2010, opioid-related overdose deaths directly increased with an increase in the prescription of opioids [71]. In 2015, opioid-related drug overdoses accounted for 33,091 deaths, approximately half involving prescription opioids [72].

Furthermore, an estimated two million people in the United States have opioid use disorder (addiction) manifest with prescription opioids, amounting to 78.5 billion dollars in economic costs every year [73]. It has now been established that the overall cost of the opioid crisis exceeds one trillion dollars. While there are a number of effective strategies available to manage chronic pain well without opioids [74], what has been earlier named “Reward Deficiency Solution System” *(RDSS)* or Precision Addiction Medicine (PAM) could assist in changing prescribing practices. Such a tool would be critical in addressing the opioid overdose epidemic and its adverse effects on the U.S. population and even across the globe [75,76]. It is challenging to provide alternative non-addicting and non-pharmacological treatments to assist in pain and addiction attenuation [77,78]. The medical establishment is encouraging the belief that alternatives with no risk or side-effects, and moderate-quality evidence supporting effectiveness, are inferior to drugs with high-quality evidence in chronic pain patients. However, a recent JAMA report provides strong evidence that non-opioid treatment such as NSAIDs fare better than chronic prescription of opioids [79,80]. 

It is noteworthy that the brain’s reward center plays an important role in modulating nociception (sensory nervous system’s response to harmful stimuli such a pain), and adaptations in dopaminergic circuitry could affect several sensory and affective components of chronic pain syndromes [81]. We should keep in mind that pain patients with analgesic tolerance should not be stigmatized as addicts unless they show evidence of inappropriate behaviors, including illicit opioid seeking and illicit analgesic doctor shopping. 

This article provides research history and findings pertaining to Reward Deficiency Syndrome (RDS). The RDS concept was first described in a general article in the American Scientist, and today over 700 articles are listed in PUBMED (5-19-19) that deal with “Reward Deficiency”. Another 1355 articles focus on “Dopamine Dysregulation”. RDS is currently found and defined in Microsoft Word and included in SAGE Encyclopedia of Abnormal Psychology and Mental Illness [1]. While a body of literature addresses the significance of dissecting the role of dopamine in “wanting” and “liking” behaviors, these concepts dovetail onto the RDS model. Understanding this umbrella term may assist in providing the rationale to propose addiction as a disease. 

The basic idea was adopted in the ASAM’s new definition of addiction in 2011 [82]. Moreover, in this article, we also provide analytic, genetic, and neurochemical evidence that could help addiction medicine and pain specialists in delivering better care, eliminating guessing (especially as it relates to becoming an addict), and providing a paradigm shift embracing “Precision Addiction Management” [76] and the induction of “Dopamine Homeostasis” [83]. This paradigm shift in the application is derived from an understanding of RDS as a disease and not moral fiber weakness. 

### 1.6. Analytics of Genetic Addiction Risk Severity (GARS) 

Possibly knowing a patient’s genetic addiction risk severity (GARS) could provide an in-depth mirror of a patient’s brain and assist in prophylactic practice, especially in early genetic identification of high risk for addiction and subsequent early intervention. The rationale behind the development of the GARS panel of reward gene polymorphisms [84] and a clinical outcome is demonstrated herein. The Brain Reward Cascade (BRC) [85] is the interaction of genes and neurotransmitters that control dopamine release. 

**Figure 1:**
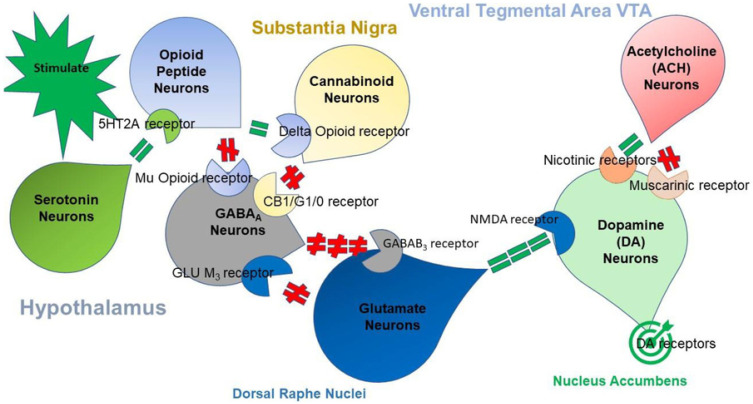
**The Brain Reward Cascade**: In the hypothalamus, environmental stimulation results in the release of serotonin, which in turn via, for example, 5HT-2a receptors activate (green equal sign) the subsequent release of opioid peptides from opioid peptide neurons the hypothalamus. In turn, the opioid peptides have two distinct effects, possibly via two different opioid receptors. One inhibits (red hash sign) through the mu-opioid receptor (possibly via enkephalin) and projects to the substantia nigra to GABAA neurons. Another stimulates (green equal sign) cannabinoid neurons (for example, the anandamide and 2-archydonoglcerol) through beta–endorphin-linked delta receptors, which in turn inhibit GABAA neurons at the substantia nigra. When activated, cannabinoids, primarily 2-archydonoglcerol, can indirectly disinhibit (red hash sign) GABAA neurons by activating G1/0 coupled to CB1 receptors in the Substantia Nigra. In the dorsal raphe nuclei (DRN), glutamate neurons can then indirectly disinhibit GABAA neurons in the substantia nigra through activation of GLU M3 receptors (red hash sign). GABAA neurons, when stimulated, will, in turn, powerfully (red hash signs) inhibit VTA glutaminergic drive via GABAB 3 neurons. Stimulation of ACH neurons at the nucleus accumbens ACH can possibly stimulate both muscarinic (red hash) or nicotinic (green hash). Finally, glutamate neurons in the VTA will project to dopamine neurons through NMDA receptors (green equal sign) to preferentially release dopamine at the nucleus accumbens (NAc), shown as a bullseye, which indicates a euphoria, or “wanting” response [86].

Figure 1 illustrates the interaction of at least seven major neurotransmitter-pathways involved in the Brain Reward Cascade (BRC). 

Variations within the BRC, whether epigenetic or genetic, may predispose individuals to addictive behaviors and altered pain tolerance. This concept has been maintained by a number of concerned clinicians and scientists that analyzed the GARS, with the first test to accurately predict susceptibility to pain, addiction, and other compulsive behaviors, which is defined as RDS [87]. Innovative strategies to contest the epidemic of opioid and iatrogenic prescription drug abuse, based on the role of dopaminergic activity in pain pathways, have been proposed [81]. Sensitivity to pain may be found in the mesolimbic projection system, where genetic polymorphisms are associated with a predisposition to pain vulnerability or tolerance [88]. Thus, they serve as unique therapeutic targets that could assist in treating pain and identify risk for subsequent addiction involving RDS and anti-reward symptomatology [89]. While we will explain our initial studies on the development of the Genetic Addiction Risk Score (GARS), which could assist in the early identification of RDS risk, including drugs and alcohol severity, it is essential to understand the complexities related to the neurogenetic basis of RDS. 

## 2. RDS-Free “Super Controls”

**Figure 2:**
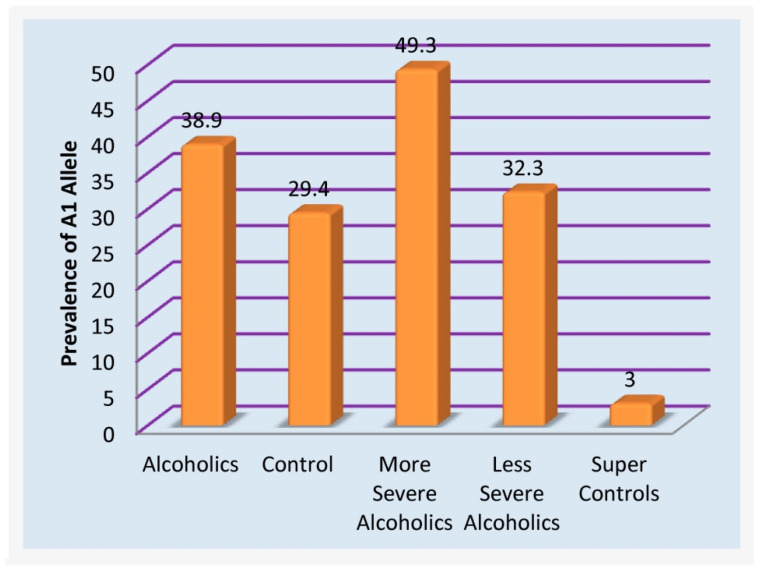
Prevalence of DRD2 A1 allele in unscreened and RDS Free controls.

At this moment, RDS controls have not been developed, and that is why simply counting risk alleles as a strategy is accepted-as verified by other investigators with non-addicting gene panels [90]. Follow-up genetic research in this area, resulting in confirmation of positive correlations with dopaminergic polymorphisms, utilizing highly-screened controls (eliminating any compulsive, impulsive, and addictive behaviors in both proband and family), may have significant consequences. In this regard, studies from Blum’s group revealed the importance of non-RDS controls [90]. In a family practice and neurology clinic in Princeton, it was determined that after using a computerized program to eliminate every potential RDS behavior in family members and the proband of 183 patients, only 30 patients were clear of any RDS behavior. The DRD2 A1 allele (known to cause a 30–40% reduction in the number of DRD2 receptors) was observed in about 33% of the unscreened population [91]. However, in the highly screened non-RDS controls, the A1 allele was found in only one patient, which translates to 3.3% [90,92] (see Figure 2). To harness appropriate genetic association studies, investigators need to ensure that hidden RDS behaviors have been systematically eliminated from their control groups; otherwise, spurious results could ensue. This concept has resulted in a disputed study published in JAMA by Yale scientists concerning the role of DRD2 allele and alcoholism whereby controls came from a French cohort of Tourette’s syndrome [93]. The consensus of the literature unequivocally establishes the importance of the DRD2 A1 allele and other associated alleles and gene loci [ANKK1] [94] with 601 articles listed in PUBMED related to alcoholism alone (1/10/21) with preponderance in favor of the risk association. 

### RDS a Phenotype

By proposing RDS as the “true” phenotype, as opposed to utilizing subtypes like Substance Use Disorder (SUD) or Behavioral Addictions (BA) that involve much more measurement error, the recovery landscape may change. Abnormal behaviors involving dopaminergic gene polymorphisms commonly reflect an insufficiency of usual feelings of satisfaction or RDS. RDS occurs as a result of a dysfunction in the “Brain Reward Cascade” (Figure 1) a complex interaction among neurotransmitters (primarily opioidergic and dopaminergic) in the brain reward circuitry [95]. Individuals with a family history of alcohol use disorder or other addictions may be born with a deficiency in the propensity to generate or utilize these neurotransmitters. Prolonged periods of stress and exposure to alcohol or other substances also can lead to a corruption of the brain reward cascade function [34], especially attenuation of endorphinergic synthesis. Blum et al. [96] assessed the possible association of four variants of dopaminergic candidate genes in RDS (dopamine transporter gene [DAT1]; dopamine D1 receptor gene [DRD1]; dopamine D2 receptor gene [DRD2]; dopamine beta-hydroxylase gene [DBH]). Blum et al. [96] genotyped an experimental group of 55 subjects obtained from up to five generations of two independent families, with multiple members affected, compared to heavily screened controls (e.g., N = 30 super control subjects for DRD2 gene polymorphisms). Data associated with RDS behaviors were collected on these subjects plus 13 deceased family members. 

**Figure 3:**
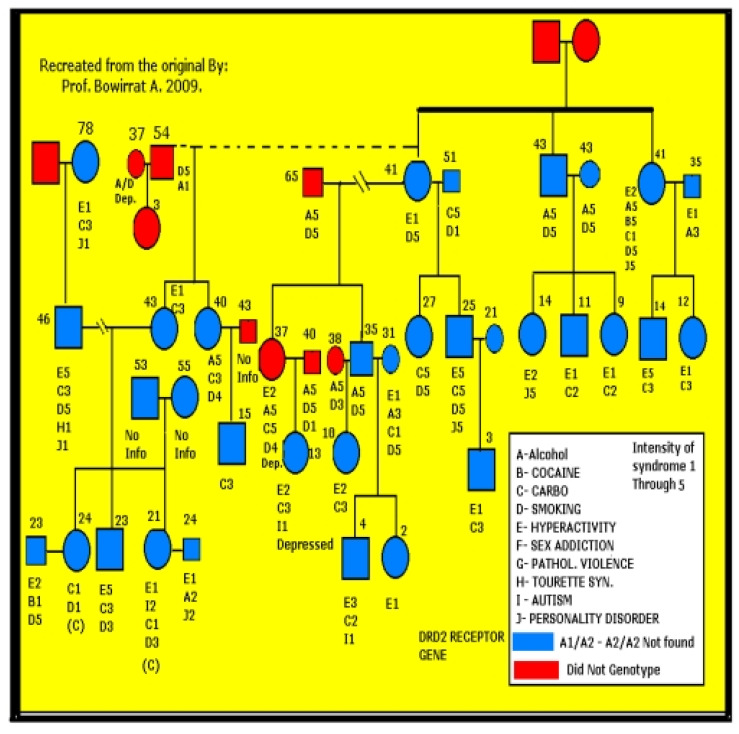
Genotypic results of the Dopamine D2 receptor gene (DRD2) polymorphism of family A (N = 32) corresponded with multiple Reward Deficiency Syndrome (RDS) behaviors [96].

Among the genotyped family members, the DAT1 and the DRD2 TaqA1 alleles were significantly (at least *p* < 0.015) more often present in the RDS families than controls. For example, 100% of Family A members (N = 32) possessed the TaqA1 allele, while 47.8% of Family B members (11/23) demonstrated expression of the allele. Significant differences were not found between the experimental and control positive rates for the other variants (see Figure 3).

Although their sample size was limited, and linkage analysis is necessary, the results reinforce the putative function of dopaminergic polymorphisms in RDS behaviors. This study exhibits the importance of a nonspecific RDS endophenotype and explains how assessing single subset behaviors of RDS may produce spurious results. The utilization of a nonspecific “reward” phenotype could be a paradigm shift in future linkage and association studies involving dopaminergic polymorphisms and additional neurotransmitter gene candidates. 

## 3. Bayes Theorem and at Birth Predictability to RDS 

In probability theory and statistics, Bayes Theorem defines the probability of an event based on prior knowledge of conditions that could be related to the event. Bayes’ theorem is referring to Reverend Thomas Bayes (1701–1761)), who first used conditional probability to provide an algorithm (his Proposition 9) that uses evidence to calculate limits on an unknown parameter, published as “An essay towards solving a problem in the Doctrine of Chances” [97]. In what he called a scholium, Bayes extended his algorithm to any unknown prior cause. Independently of Bayes, Pierre–Simon Laplace, in 1774 and later in his 1812 “Théorie Analytique Des Probabilités”, used conditional probability to formulate the relation of an updated posterior probability from a prior probability, given evidence. Sr Harold Jeffreys put Bayes’ algorithm and Laplace’s formulation on an axiomatic basis. Jeffreys wrote that Bayes’ theorem “is to the theory of probability what the Pythagorean theorem is to geometry”. We used this mathematically based theorem to predict the chance that if you carry the DRD2 A1 allele at birth: what is the Predictive Value (P.V.) that the individual would potentially indulge in drug and non-drug behavioral addictive behaviors (RDS)? 

The dopaminergic system, particularly the dopamine D2 receptor, has been profoundly implicated in reward mechanisms in the mesolimbic circuitry of the brain. Dysfunction of the D2 dopamine receptors contributes to an aberrant substance-seeking behavior (i.e., alcohol, drug, tobacco, and food). Decades of research indicate that genetics play an important role in vulnerability to severe substance-seeking behavior. Blum et al. [98] and Archer et al. [99] proposed that variants of the D2 dopamine receptor gene are important common genetic determinants in predicting compulsive disease. Blum et al. [100] determined through the Bayes approach that when they added up many RDS behaviors and applied the Predictive Value (P.V.), they found a 74.4% value. This leads to the unfortunate fact that a newborn with the DRD2 variant (A1 compared to A2 [usual]) will have a 74 % chance of developing RDS behaviors and could shift to addiction. In this regard, the full GARS panel (to be explained below) has not yet been analyzed using Bayes Theorem, but we are very confident that the P.V. would even be higher.

## 4. Understanding GARS

Blum’s group and others have published considerably on the neurogenetics of brain reward systems, particularly on the genes related to dopaminergic function [101,102,103]. Blum coined “Reward Deficiency Syndrome” (RDS) to portray behaviors found to have a gene-based association with hypodopaminergic function [104]. RDS as a concept has been embraced in many subsequent studies to increase our understanding of addictions and other impulsive, compulsive, and obsessive behaviors. Interestingly, in one published study, Blum’s group was able to detail lifetime RDS behaviors in a recovering addict (17 years sober) blindly by evaluating resultant Genetic Addiction Risk Score (GARS) data only [105]. It was hypothesized that genetic testing at an early age might be an effective preventive strategy to decrease or eliminate pathological behavioral and substance-seeking activities [106], providing further support of the disease model. Here, we consider a particular number of genes, their polymorphisms, and associated risks for RDS. While utilizing GWAS, there is evidence for convergence to reward candidate genes [107]. The evidence presented in many studies serves as a credible brain-print, providing relevant genetic information that will bolster targeted therapies to improve recovery and prevent relapse on an individualized basis [108]. The leading driver of RDS is a hypodopaminergic trait (rooted in genes) as well as additional epigenetic states (deacetylation and methylation on chromatin structure) associated with transgenerational effects of maternal depression and addiction [109]. As David E. Smith (2017) [110] points out, we have entered a new era in addiction medicine that adopts the neuroscience of addiction and RDS as a pathological condition in the brain reward circuitry that calls for appropriate evidence-based therapy and early genetic diagnosis. 

**Figure 4:**
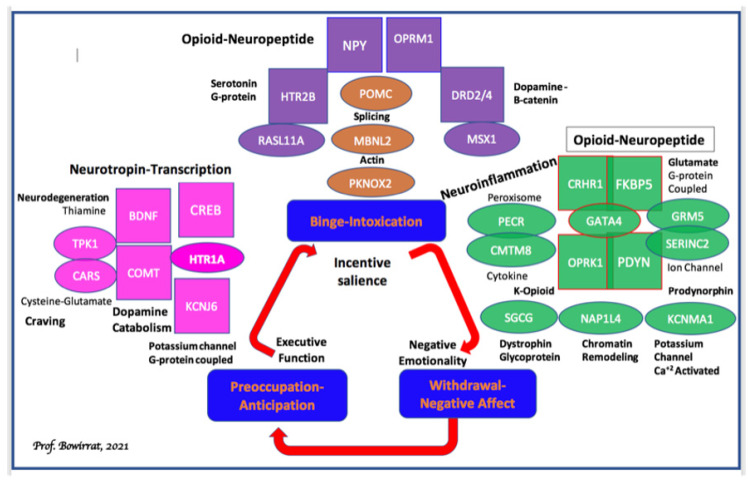
**Candidate genes for alcohol dependence characterized into stages of the addiction cycle**: This schematic shows the three stages of the addiction cycle: (1): binge-intoxication (blue), (2): withdrawal-negative affect (red), and (3): preoccupation-anticipation (green); also shown are behavioral domains linked to each stage of the cycle: incentive salience, negative emotionality, and executive function, respectively. Candidate functional genes identified by non-GWAS approaches are shown in ovals and colored coded according to the relevant addiction stage the gene is hypothesized to function. GWAS candidates are shown in rectangles color-coded according to the relevant addiction stage the gene is hypothesized to function. The general biological function of each gene in the figure is highlighted in black. Non-GWAS functional gene relationships are indicated by overlapping ovals. Hypothesized functional relationships between non-GWAS genes and GWAS candidates are indicated by overlapping ovals and rectangles. GWAS candidates in each stage are grouped according to potential biological functional similarities where possible. Finally, one example of pleiotropy is shown in the preoccupation-anticipation stage. BDNF-COMT functional variants have pleiotropic effects in both the preoccupation-anticipation stage and the withdrawal-negative affect stage [112].

Pharmacogenomic testing of candidate genes such as dopaminergic, endorphinergic, cannabinoid, glutaminergic, GABAergic, receptors, serotonergic and dopamine transporters, and catabolic enzymes of Mono-Amine–Oxidase (MAO-A) and Catecholamine–Methyl-Transferase (COMT), and many others seem prudent but is controversial [111]. However, Koob’s group [112] at NIAAA has provided evidence for multiple reward gene association studies, which show significant associations with alcohol use disorder (Figure 4).

Understanding these concepts will advance pharmacogenomics, personalized solutions, and clinical outcomes. Genetically classifying the risk for all RDS behaviors, particularly in compromised populations (e.g., African–Americans and poverty populations), may be a frontline tool to assist municipalities in providing better resource allocation [113]. The GARS test predicts the risk for RDS behaviors shared with the Addiction Severity Index—a clinical predictive test. It is essential to recognize that GARS cannot show false positives since it assesses an entire panel of gene polymorphisms predicting alcohol and drug severity as a cluster [114]. This kind of testing does not appropriately allow for performing a Receiver Operator Curve (ROC) analysis. 

Blum’s group, in conjunction with Geneus Health and Colorado University, in unpublished research (a 5-year sojourn), wanted to address the genetic risk for drug/alcohol seeking by evaluating the shared effect of polymorphisms for reward genes [a genetic addiction risk score (GARS) of eleven polymorphisms and 10 genes] contributing to a hypodopaminergic-trait. This trait profile was associated with RDS-related substance abuse risk. The patient population utilized 393 poly-drug abusers attending 8 independent treatment centers from the United States. Clinical severity of drug and alcohol use behaviors was assessed using the Addiction Severity Index (ASI-MV). A cheek cell sample for DNA genotyping was acquired from N = 273 (from seven centers) combined with ASI phenotype. The average age of the analysis sample was 35.3 years (S.D.–13.1, Range: 18–70), of which 88.1% (N = 244) self-reported their race as White and 57.8% (N = 160) were male. Among the patient population, N = 393, 1.5%, 80.7%, and 17.6% scored in the high, moderate, and low severity range, respectively. The mean number of GARS alleles was 7.97 (S.D. = 2.34) and ranged between 3 and 17 alleles. Every measured genotype was in Hardy-Weinberg Equilibrium (HWE). Initial evaluation of the relationship between the GARS genotype panel and the Alcohol Risk Severity Score (using the Fishers Exact Test) demonstrated a significant predictive relationship (Χ2 = 8.84, *p* = 0.004, df = 1, 2-tailed) that remained significant after controlling for age (*p* < 0.01). A similar, though less strong, relationship was acquired from linear regression (b = −0.122, t = −1.91, *p* = 0.10, 2-tailed) and chi-square (*p* = 0.05) analyses of the ASI Drug Severity Risk Score. Correcting this result along a priori lines showed a *p*-value of 0.05 (1-tailed) for the association between the ASI Drug Severity Risk score and the GARS panel. This result, albeit significant, is less robust for drugs compared to alcohol. It is to be noted that we are comparing a paper-pencil, self-reported test with an objective genetic test. Since psychoactive drugs are illegal, there could be a number of patients lying about their use. In fact, it is known that carriers of the DRD2 A1 allele as measured by GARS show an association with a higher Defense Style Questionnaire than non-carriers, as reported by Comings et al. [115]. 

The test results reveal that if a patient harbors any combination of 4 GARS risk alleles, then it is predictive of drug severity (*p* < 0.05). Any combination of 7 GARS risk alleles is predictive of alcohol severity (*p* < 0.004); 100% of the patients from chemical dependency treatment programs carry at least one risk allele. 

In fact, the higher the number of risk alleles, the stronger the prediction of alcohol or drug use severity. It was also found that family problems, psychological issues, and medicalization significantly correlated as well. One notable caveat was that if they changed any specific SNP, the significance was lost. This speaks to the question of counting alleles vs. odds ratios and real non-RDS controls. Also, when they multiply a fixed number to provide power to any gene in the GARS, the significance is lost. This strongly suggests the importance of the selection of the alleles in the GARS panel. Any deviation will produce false results that may occur with other tests that have little to no clinical research to validate their test results, especially if the test is based on spurious controls that are not RDS free [96], despite claims. 

**Figure 5:**
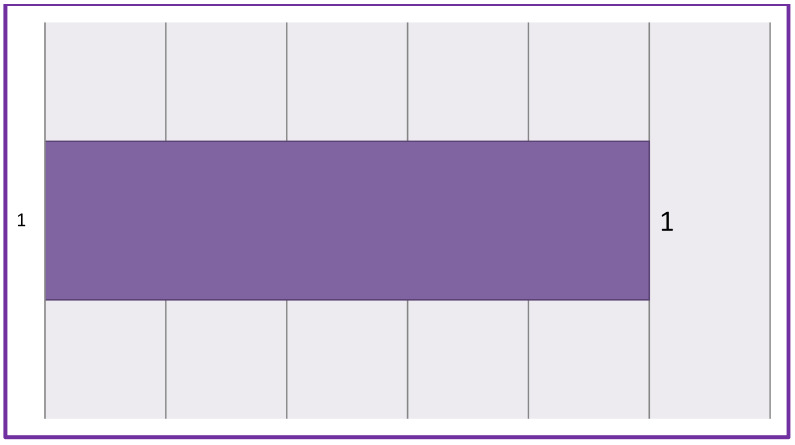
GARS Panel Association Studies [85].

To help understand the rationale for the selection of the reward genes in the GARS panel, we provide a graphic representation of the number of association studies listed in PUBMED as of 11-12-17. Most association studies analyze both case controls and disease phenotype (see Figure 5).

As stated earlier, the GARS report is a restricted cluster of gene polymorphisms that predicts both drug and alcohol severity based on the Addiction Severity Index (ASI). It is not specific to any drug per se but to an array of RDS substance and non-substance addictive behaviors (e.g., overeating, pathological gambling, gaming, internet addiction, shopping, hoarding, sex addiction, etc.) The interesting concept of RDS is that the common genetic rubric is hypodopaminergia that predisposes individuals to all addictive behaviors, including “liking and wanting” [103]. In an attempt to resolve controversy related to the causal contributions of mesolimbic dopamine (DA) systems to reward, we examine the three main competing explanatory categories: “wanting”, “learning”, and “liking”. That is, dopamine may mediate (a) the pursuit of rewards by attributing incentive salience to reward-related stimuli (wanting), (b) learned predictions about rewarding effects (learning), or (c) the hedonic impact of reward (liking). We examine these hypotheses, particularly as they relate to Reward Deficiency Syndrome (RDS). We observe that most of the evidence supports the incentive salience or “wanting” hypothesis of Dopamine function. Neuroimaging studies have revealed that drugs of abuse, palatable foods, and anticipated behaviors such as sex and gaming impact brain regions involving reward circuitry and may not be unidirectional [116]. 

Drugs of abuse enhance dopamine signaling and sensitize mesolimbic mechanisms that evolved to attribute incentive salience to rewards. Addictive drugs share common features, including that they are voluntarily self-administered, they enhance (indirectly or directly) dopaminergic synaptic function within the nucleus accumbens (NAC), and that they stimulate the functioning of the reward circuitry (generating the “high” that drug users pursue). Although initially believed simply to encode the set point of hedonic tone, these circuits now are believed to be functionally more complex, also encoding attention, reward expectancy, disconfirmation of reward expectancy, and incentive motivation. Elevated stress levels, together with polymorphisms of dopaminergic genes and other neurotransmitter genetic variants, may have a cumulative effect on vulnerability to addiction. The RDS model of etiology holds very well for a variety of chemical and behavioral addictions. The reduced net release of dopamine in the reward center of the brain (N. Accumbens) is indeed the culprit. It is well known from many metabolic/pathophysiology studies from both NIDA and NIAAA that “Dopamine Homeostasis” is a laudable goal, and it is the combined result of genetics (DNA) and epigenetics (environmental components) [67,104]. In terms of case-control data relating to GARS and allelic association for the risk, we present Table 1 for readership scrutiny.

### 4.1. Do Stress and Social Influences Alter Cell Function and Lead to Addictive Behaviors Epigenetically?

Substance use disorders (SUDs) are highly prevalent. SUDs involve vicious cycles of binges, followed by occasional periods of abstinence and recurrent relapses. The relapses occur despite treatment and adverse psychosocial and medical consequences. There is compelling evidence that early and adult stressful life events are risks factors for the development of addiction; these stressful life events can serve as cues that trigger relapses. However, the fact that not all individuals who face traumatic events develop an addiction to illicit or licit drugs suggests the existence of individual and familial factors that afford resilience and protect these mentally healthy individuals. Understanding the epigenetics of responses to stressful events and the administration of drugs of abuse provides the basis for developing novel treatments. Our understanding of the importance of stress and resultant epigenetic insults onto the brain’s neurochemistry, as wells mRNA directed gene expression, is one aspect of why we believe that it is difficult to simply abstain from unwanted horrific drug dependence.

Moreover, the psychobiology of resilience and alterations in epigenetic markers that have been observed in models of resilience may assist in finding therapeutic targets. One laudable goal is to accept the fact that there are genetic antecedents to addictive behaviors, in spite of the disbelievers, and as such, help direct novel approaches for treatment and even prevention. Accordingly, the possibility that addiction treatment should involve pharmacological/nutraceutical DNA-guided precision approaches that increase resilience in at-risk individuals requires continued intensive research. Similar approaches can also be used with patients who have already succumbed to the terrible effects of addictive substances.

Blum et al. [117] investigated the effects of an amino acid (precursors to neurotransmitters and enkephalinase inhibition) and vitamin mixture on an inpatient, chemically-dependent subject pool to examine the role of neurotransmitters in facilitating recovery and readjustment to a detoxified, sober state. KB220 is developed from amino acids that are precursors for neuromodulators and neurotransmitters thought to be involved in drug and alcohol-seeking behavior. In a placebo-controlled, double-blind, randomized study of 62 alcoholics and polydrug abusers, KB220 patients had a significantly decreased stress response, as determined by the skin conductance level (SCL). In addition, significantly improved BESS Scores (behavioral, emotional, social, and spiritual) and Physical Scores were also observed. After detoxification, there was a six-fold reduction in AMA rates when comparing KB220 vs. placebo groups. In this inpatient treatment experience, KB220 facilitated the recovery rate. It permitted patients to respond more quickly and thoroughly to the behavioral goals of the program, as measured by the BESS Score. The use of KB220 to attain enkephalinase inhibition and precursor amino acid loading allows a practitioner to restore the neurochemical changes that occur in drug abuse and alcoholism. These findings increase our understanding of the clinically relevant neurobiological mechanisms which underlie compulsive disease.

Prenatal stress (PNS) is a well-established phenomenon that generates perturbations in nervous system development, resulting in behavioral alterations that include hyperresponsiveness to stress, novelty, and psychomotor stimulant drugs (e.g., cocaine, amphetamine). A large body of information exists to suggest that depression in female offspring continues through adulthood. This is related to a decrease of dopaminergic neurotransmission at the NAc, and the present data offers new evidence in support of the hypothesis that maternal stress during gestation increases the risk of depression in the offspring [118]. Moreover, PNS animals display enduring alterations in basal ganglia and cocaine-induced changes in glutamate and dopamine transmission in limbic structures, which exhibit pathology in alcoholism and drug addiction. This suggests that these alterations may contribute to an increased propensity to self-administer large amounts of drugs of abuse or to relapse following periods of drug withdrawal. Given that cocaine and alcohol have actions on common limbic neural substrates (albeit by different mechanisms), it has been hypothesized that PNS would elevate the motivation for and consumption of alcohol. Accordingly, Campbell et al. [119] have found that male C57BL/6J mice subject to PNS exhibit higher operant responses and consume more alcohol during alcohol reinforcement as adults. Alterations in glutamate and dopamine neurotransmission within the forebrain structures appear to contribute to the PNS-induced predisposition to high alcohol intake and are induced by excessive alcohol intake. 

### 4.2. Does Long-Term Excessive Drinking Alter Cell Function and Lead to Addiction?

Systemic administration of agents that (1) increase dopamine (DA) or synaptic levels of serotonin (5-HT); (2) activate 5-HT1A, 5-HT2, D2, D3, or GABA(A) receptors; or (3) block opioid and 5-HT3 receptors decrease ethanol/drugs intake in most animal models. Neuropharmacological, neuroanatomical, and neurochemical studies indicate innate differences exist between the low alcohol-consuming and high alcohol-consuming rodents in various CNS limbic structures. Additionally, reduced mesolimbic DA and 5-HT function have been noted during alcohol withdrawal in common stock rats. Depending on the animal model in question, the abnormalities in the mesolimbic dopamine pathway and/or the serotonin, opioid, and GABA systems that regulate this pathway may underlie vulnerability to the abnormal alcohol-seeking behavior in the genetic animal models [120]. As mentioned above, in terms of stress-induced alcoholism, our laboratory developed a schema to understand the relationship between genetics and environmental etiological indices for drug craving behavior (inability to stop). The consensus of the literature points towards a neuro-psycho-genetic model of alcoholism. Evidence in both animals and humans tends to support the proposed “genotype” theory of alcohol-seeking behavior, whereby a predisposition to alcohol preference may be mediated in part by either innate (genetic) or environmentally (stress and/or alcohol) induced brain opioid peptide dysfunction [121].

Scrutiny of the data from a series of studies performed by Blum’s group reveals that the C58/J alcohol-preferring mice have significantly reduced baseline methionine-enkephalin levels in both the hypothalamus and corpus striatum compared to C3H/CHRGL/2 non-alcohol-preferring mice. For these two strains, analysis of methionine-enkephalin levels in brain regions, particularly the pituitary, amygdala, midbrain, and hippocampus, did not show any significant differences. This suggests that the hypothalamus may indeed be a specific locus involved in regulating alcohol intake via the molecular interaction between neuro-amines, opioid peptides, as they are influenced by genetics and environment [122]. Additionally, Blum et al. [123] investigated ethanol preference in both the C57Bl/6N and C57Bl/6J mice utilizing the three-choice 2-bottle preference test. In addition, these sub-lines were evaluated for whole-brain methionine-enkephalin levels, which were significantly reduced in C57Bl/6J mice (alcohol-preferring) compared to C57Bl/6N mice (alcohol non-preferring). These findings support the involvement of the peptidyl opiates in ethanol-seeking behavior [123]. This work was followed up by showing a negative correlation between the amount of ethanol (10%) consumed and endogenous levels of the brain [Met]enkephalin in C57BL/8 wk. These results further suggest that the endogenous brain peptidyl opiates may play a crucial role in alcohol-seeking behavior [124].

These experiments led Blum’s group to explore the potential therapeutic rationale involving the utilization of novel inhibitors of carboxypeptidase A (enkephalinase), which raises endogenous enkephalin levels and possesses anti-alcohol seeking effects [122]. In fact, they have been able to significantly attenuate both volitional and forced ethanol intake respectively by acute and chronic treatment with hydrocinnamic acid (a metabolite of D-phenylalanine) and D-phenylalanine, which are known carboxypeptidase (enkephalinase) inhibitors. Since these agents, through their enkephalinase inhibitory activity, raise brain enkephalin levels, Blum et al. [122] proposed that excessive alcohol intake can be regulated by alteration of endogenous brain opioid peptides. This finding may have great relevance as a “*pharmacogenetic engineering*” tactic to block unwanted (against will) craving behavior for alcohol, drugs, and even food. 

The question of nature vs. nurture in terms of substance-seeking behavior has been addressed in many studies across the globe. We must be cognizant that it is always the relationship between both the environment and our genome that predicts any behavioral outcome: P = G + E. Recently, Campbell et al. [119] reviewed problematic aspects of alcohol abuse disorder (excessive alcohol consumption) and the inability to refrain from alcohol consumption during attempted abstinence (loss of will-power during recovery). While being cognizant of the significant role of genetics (up to 50% contribution of the variance) they concluded that early environmental trauma alters neurodevelopmental trajectories that can predispose an individual to substance abuse and numerous neuropsychiatric disorders. Indeed, the above evidence and many other studies gleaned from the scientific literature provide strong indisputable biogenetic data to support the disease model of all addictive behaviors [125]. In fact, the extant literature confirms that an array of polymorphic genes associated with neurotransmitters and second messengers regulate the net release of dopamine in the Nucleus Accumbens (NAc) in the mesolimbic region of the brain. They are linked primarily to motivation, anti-stress, incentive salience (wanting), and wellbeing. Notably, the Nobel Prize was awarded in 2000 to Carlsson, Greengard, and Kandel for their work on the cellular and molecular function of dopaminergic activity in neurons. This historical psychopharmacological work involved neurotransmission of dopamine, endorphins, glutamate, and serotonin; the historical work also involved the seminal work of Blum, Volkow, Gold, Nestler, and others exploring neurotransmitter function and associated behaviors. Currently, American citizens are facing their second and worst opioid epidemic. Opioid prescriptions and easy access drive this epidemic of overdoses and opioid use disorders (OUDs). At this point in time, the clinical consensus is to treat OUDs as if they were an opioid deficiency syndrome, utilizing long-term to life-long opioid substitution therapy. Opioid agonist administration is understood as necessary to replace missing opioids, treat OUD, and prevent overdoses, akin to how insulin is used to treat diabetes. Treatment of addiction and OUD, in general, is similar to the endocrinopathy conceptualization in that it regards opioid agonist MATs as an essential core to therapy. Is this approach logical? Other than as a form of harm reduction, is using opioids to treat OUD therapeutic or harmful in the long term? 

To underscore the fact that alcohol significantly reduces the synthesis of endorphins, Blum’s group provided strong evidence [123]. Golden Syrian hamsters were individually placed in cages with three drinking bottles--one empty, one containing water, and the third containing water and ethanol. Control hamsters received water only. After one year (approximately 20 years of active street drinking in humans), the experimental hamsters showed a significantly reduced concentration of a leucine-enkephalin-like immunoreactive substance in the basal ganglia compared to the control hamsters. This finding suggests that the action of ethanol involves endogenous peptidyl opiates. In addition, chronic administration of morphine to rats for four weeks resulted in a 50–60% decrease in the tissue concentrations of beta-endorphin and in the in vitro release from the neuro-intermediate pituitary. Thus, long-term treatment with morphine appears to depress beta-endorphin formation in the intermediate rat pituitary at the pre-translational level by markedly decreasing the activity of mRNA coding for the beta-endorphin/ACTH precursor without any alteration in the processing of this precursor [126].

### 4.3. Anti-Reward Symptomology

Converging lines of evidence indicate that the pathophysiology of pain and drug-seeking is mediated to a large degree via allostatic neuroadaptations in reward and stress-related brain circuits. Thus, reward deficiency (RD) represents a within-system neuroadaptation to drug-seeking-induced protracted activation of the reward circuits that results in depletion-like hypodopaminergia, clinically manifested anhedonia, and diminished motivation for natural reinforcers. Anti-reward (AR) conversely pertains to a between-systems neuroadaptation involving over-recruitment of essential limbic structures (e.g., the basolateral and central amygdala nuclei, the lateral tegmental noradrenergic nuclei of the brain stem, the bed nucleus of the stria terminalis, the habenula, and the hippocampus) responsible for a massive outpouring of stressogenic neurochemicals (e.g., norepinephrine, corticotropin-releasing factor, vasopressin, hypocretin, and substance P), giving rise to negative affective states such as anxiety, fear, and depression. Borsook et al. [127] propose here the Combined Reward deficiency and Anti-reward Model (CReAM), in which biopsychosocial variables are modulating brain reward, motivation, and stress mechanisms can interact in a ‘downward spiral’ fashion to exacerbate the intensity, chronicity, and comorbidities of chronic pain syndromes (i.e., pain chronification) and reward processing linked to aberrant drug and non-drug seeking behaviors. 

### 4.4. Future Perspectives

It is now known that nature (genes) combined with nurture (environment), and resulting behavioral outcomes within *Homo Sapiens*, that the contribution is approximately 50% genes and 50% epigenetics (environmental influence on genetic expression), especially in alcohol use disorder [128,129]. Thus, molecular genetics or DNA testing is fundamental, especially linking aberrant behaviors to any individual. Blum et al. [103] proposed that any disturbance along the reward cascade that might be due to either gene variations (polymorphisms) and/or environmental influences (epigenetics) can result in various aberrant and addictive behaviors (i.e., RDS). Despite a continued global-wide search to divulge specific candidate genes or clusters characterized from high-density SNP arrays, it is common knowledge that many attempts have failed to replicate and/or been inconclusive. However, Palmer et al. [130] recently showed that between 25–36% of the genetic variance in the generalized vulnerability to substance dependence might be attributable to common single nucleotide polymorphisms.

Moreover, the additive effect of common single nucleotide polymorphisms is shared across principal indicators of various comorbidities. Furthermore, as a result of such research studies, recent evidence has shown that specific gene variants may account for risk prediction. As mentioned above, adopting a new approach, a Bayesian approach from earlier studies from Blum’s laboratory [98] concluded that a Positive Predictive Value (PPV) of 74%, specifically for the DRD2A1 allele, appeared to be an indication that if a child is born with this polymorphism, then he/she will have a much higher risk of future addictions (i.e., drugs, food, or aberrant behaviors) at some point in their lives. Since the 1990 finding of the DRD2 gene association of the *Taq A1* allele and severe alcoholism, laboratories all across the globe, including NIDA and NIAAA, not only confirmed this early work but also extended the importance of various candidate genes, specifical genes for second messengers in the reward system [112]. 

An example is Moeller et al. [131], who suggested that drug cues contribute to relapse. Their neurogenetic results have identified the DAT1R 9R allele as a vulnerability allele for relapse, especially during early abstinence (e.g., detoxification). The DAT1R 9R allele influences the fast-acting transport of dopamine, sequestered from the synapse, leading to a hypodopaminergic trait. It is important to use genetic testing to uncover reward circuitry gene polymorphisms, particularly those linked to dopaminergic pathways, including opioid receptor(s), as a method of attaining better treatment results. Understanding the relationship between the reward circuitry’s participation in buprenorphine outcomes and corresponding genotypes delivers an innovative model to enhance a patient’s clinical experience and improved relapse prevention during opioid replacement therapy. While there are other genetic proposed panels especially linked to OUD, none have provided evidence for a genetic addiction risk severity (GARS) of known risk polymorphisms of reward genes associated with an increased genetic risk for addiction and other RDS behaviors based on analytic evidence such as ASI prediction of drug and alcohol severity as well as utilization of an unpublished newly validated RDS index developed with our Hungarian associates (Demetrovics and Blum, 2021 personal communication). 

As proposed previously, activation, rather than blocking mesolimbic dopaminergic reward circuitry, in the long-term treatment of RDS is the preferred modality [132]. Although the acute treatment may consist of antagonism of postsynaptic NAc dopamine receptors (D1-D5), long-term treatment should consist of activating the DA system, such as the release and activation of DA in the NAc. This proposed theory suggests that an addict’s excessive craving behavior is attributed to the reduction in the number of D2 receptors, which can affect carrying the DRD2 A1 allelic genotype. In contrast to this, a standard or sufficient density of D2 receptors results in reducing craving. Thus, a primary goal of treatment and preventing such substance use & misuse, and even non-substance addictive behaviors including obesity, pathological gambling, sex addiction, ADHD, shopping and hoarding, etc. [133], might be to induce proliferation of D2 receptors in individuals with the genotype making them genetically vulnerable. While in vivo experiments that used a typical D2 receptor agonist induce down-regulation, in many in vitro experiments, results have demonstrated that notwithstanding genetic antecedents, the constant stimulation with a known D2 agonist, such as bromocriptine, often results in significant proliferation of D2 receptors in the Dopamine system [134]. However, chronic treatment results in down-regulation instead of up-regulation or balance, as is proposed for KB220Z [135]. That is a reason for failure in treatment with potent D2 agonists. The work of Thanos and associates employing gene therapy with a CDNA construct of the DRD2 gene resulted in significant attenuation of both ethanol and cocaine-seeking behaviors in genetically preferring rodents [136,137,138]. 

Finally, we propose that Precision Addiction Management (PAM) is the next generation genetically based approach to achieve required dopamine homeostasis via customized pro-dopamine regulation, as well as early identification and the critical enablement of early intervention therapy. This new dopaminergic regulatory approach seems more prudent than to continue to “lock people into an addiction to potent opioids–like molecules” rather than finding ways to ameliorate a potential hypodopaminergia (a root cause of all addictive behaviors), thereby combating not only the opioid crisis but all RDS non-substance addictive behaviors as well. We must keep in mind that while childhood internet gaming or even smartphone addiction may seem innocuous, it provides a clue to the family that this behavior must be taken seriously because this infectious behavior, if unrecognized, could indeed result in another fatality due to unwanted fentanyl overdose in our loved ones. The best medicine is to embrace the concept of “Love Your Pups”, and by unraveling genetic risk, stop the guessing and join the 21st century of the genome and by doing so prevent anhedonia or redeem joy once again with concomitant enhanced quality of life [139]. 

It is noteworthy that tobacco smoke may be a gateway to other drugs of abuse, including marijuana use, especially in children with RDS-cocaine abuse [140]. It has been argued that among current college smokers, 40% started smoking by learning to inhale marijuana and then started using tobacco, or they started using tobacco at the same time [141]. The conundrum here is, without a clear diagnosis of early-onset SUD, it is difficult to ascribe the concept of the gateway theory generalized to any specific drug because of the abuse of any particular psychoactive drug, such as marijuana or tobacco, may occur with or without the RDS-phenotype. However, gene testing, as we propose, will provide additional and pertinent information regarding the connection between RDS-symptomatology, and SUD, including marijuana, tobacco use, and other risk behaviors. More importantly, existing polymorphisms could then be targeted to provide personalized medicine to the individual child, particularly in carriers of the *DRD2* A1 allele (among other gene polymorphisms). According to Volkow et al. [142], in support of our RDS concept: 

Behaviors such as eating, copulating, defending oneself or taking addictive drugs begin with a motivation to initiate the behavior. Both this motivational drive and the behaviors that follow are influenced by past and present experience with the reinforcing stimuli (such as drugs or energy-rich foods) that increase the likelihood and/or strength of the behavioral response (such as drug-taking or overeating). At a cellular and circuit level, motivational drive is dependent on the concentration of extra-synaptic dopamine present in specific brain areas such as the striatum. Cues that predict a reinforcing stimulus also modulate extra-synaptic dopamine concentrations, energizing motivation. Repeated administration of the reinforcer (drugs, energy-rich foods) generates conditioned associations between the reinforcer and the predicting cues, which is accompanied by downregulated dopaminergic response to other incentives and downregulated capacity for top-down self-regulation, facilitating the emergence of impulsive and compulsive responses to food or drug cues. Thus, dopamine contributes to addiction and obesity through its differentiated roles in reinforcement, motivation and self-regulation, referred to here as the ‘dopamine motive system’, [AKA also RDS], which, if compromised, can result in increased, habitual and inflexible responding. Thus, interventions to rebalance the dopamine motive system might have therapeutic potential for obesity and addiction. 

It is important to recognize that post drug abuse or non-drug addictive behaviors and the resultant endophenotype include many epigenetic insults affecting the brain reward circuitry. These abnormalities require a wide range of tertiary therapeutic approaches. These known insults include but are not limited to: aberrant craving behaviors, neurotransmitter deficits, cognitive and memory declines, focus, high stress, helplessness, fear, loss of control, loss of self–esteem, economic factors, harm avoidance behavior, physiological dysfunction like abnormal P300, electrophysiological insults, altered resting-state functional connectivity, poor communication skills, negative mindfulness, suicidal ideation, anhedonia, sexual dysfunction, hormonal imbalance (including high Corticotrophin releasing Factor (CRF release), metabolic, and even cardiovascular issues. While genetic addiction risk testing coupled with pro-dopamine regulations has important heuristic value, treatment must include various modalities. It is equally important to recognize the importance of highly sophisticated brain mapping tools like rsfMRI, SPECT, QEEG, PET, TOVA testing, as routine as cardio checkups (see Table 1). 

Table 1 lists potential Brain Scan tools that may enhance brain diagnostic protocols in primary care medicine.

**Figure 6:**
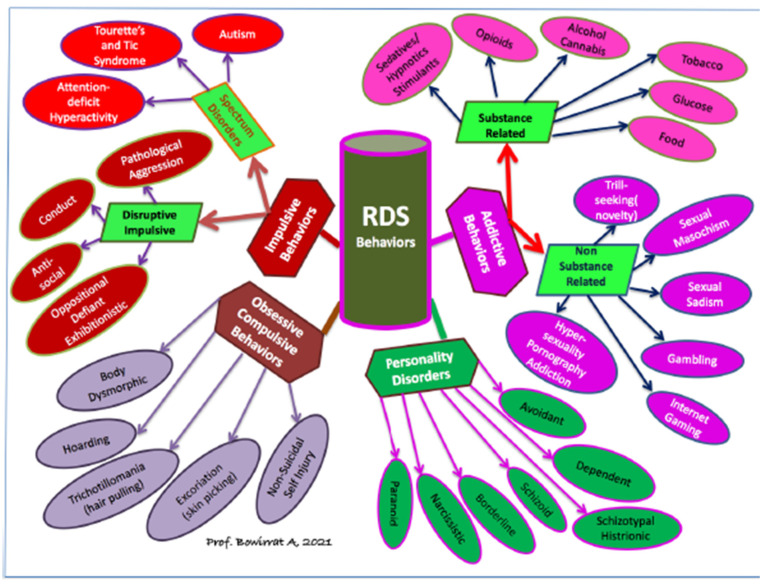
The Disease Model of Reward Deficiency Syndrome (RDS).

The following disease model of both drug and non-drug behaviors supported by a plethora of thousands of peer review studies is represented in Figure 6.

## 5. Conclusions

While Reward Deficiency Syndrome (RDS) refers to both drug and non-drug addictive behaviors, one major concern globally, but particularly in the United States of America, is the current opioid crisis. Opioid use disorders and overdoses are an epidemic. Opioid deaths, opioid overdoses, substance use, depression, anhedonia, and suicides have combined to produce so many deaths of despair that the life expectancy in the USA is declining [2]. From 1999 and 2010, deaths from opioid-related overdoses increased significantly in parallel with increased prescriptions of opioids. Opioid-involved drug overdoses accounted for 92 thousand deaths in 2020. Now that fentanyl has replaced prescription opioids; deaths are more common.

Furthermore, an estimated 2 million individuals in the United States have opioid use disorder related to prescription opioids, accounting for 78.5 billion dollars in economic costs every year. In the United States, every seventeen minutes, one person overdoses. It is now well-established that the overall cost of the opioid crisis is greater than one trillion dollars. Medication-assisted treatments for OUDs, such as methadone, buprenorphine, and suboxone, are the most prescribed medications for patients with OUDs [143]. Sadly, most patients drop out early, and few follow the advice of their treatment professionals. Slips, relapses, and overdoses quickly follow MAT discontinuation, but the patients are simply prescribed the same treatment in perpetuity [144]. With newer approaches [86], the opioid antagonist Naltrexone may also find a place in the MAT treatment algorithm. Opioid prescribing for non-malignant pain, dental problems, and even fractures have been curtailed as physicians are writing fewer opioids for pain. However, that has not stopped the fentanyl manufacturers and distributors. They have taken over prescription opioids and heroin as the number one opioid abused today. 

While there are several valid strategies available to manage chronic pain effectively without opioids (e.g., Transcranial magnetic stimulation [TMS] and H-Wave therapy), most agencies agree that, as a collective community, we are being tested to produce alternative non-addicting and non-pharmacological alternatives to assist in addiction and pain attenuation. The medical establishment is now encouraging alternatives with no risk or side-effects and moderate-quality evidence, supporting Non-Steroidal Anti-inflammatory Drugs (NSAIDs) in chronic pain patients. A recent JAMA report provides strong evidence that non-opioid treatments fare better than chronic opioids. It is noteworthy that the reward center of the brain plays a key role in the adjustment of nociception and that adaptations in dopaminergic circuitry may impact several sensory and affective components of chronic pain syndromes, keeping in mind that pain patients that present with analgesic tolerance should not be stigmatized as being addicts unless they show evidence of inappropriate behaviors including illicit opioid seeking and licit analgesic doctor shopping. Possibly knowing a patient’s genetic addiction risk severity (GARS™) could help provide an in-depth mirror of a patient’s brain and assist in prophylaxis, especially in early genetic identification of high risk for addiction. In this article, we are providing analytic, genetic, and neurochemical evidence that could help addiction medicine and pain specialists in providing better care and eliminating guessing, especially as it relates to becoming an addict and providing a paradigm shift, embracing PAM and the induction of “Dopamine Homeostasis”. 

We argue herein that if key-opinion leaders continue to promote opioids to tertiary treat opioid dependence without developing epigenetic manipulation of brain neurochemistry to induce homeostasis across brain circuitry, we are setting up for dismal failure. The argument against the disease model of addictive behaviors is flawed. However, the missing link is not just related to opioid use disorder but to the underpinnings of all addictions, including non-substance behavioral addictions. Understanding the rubric of RDS in this context certainly provides compelling arguments for the entire scientific community to explore non-addicting ways to target specific genetic antecedents with the primary goal to achieve “dopamine homeostasis”. With this stated, it is clear that because of transfer from one addiction to another, while our opioid epidemic is receiving most of the media attention, to provide Homo Sapiens with a life free of the chains of addiction, we must begin to identify addictive-like risk as early as possible. 

Up until now, that would not have been possible. However, as depicted in this article, the authors provide a framework to accomplish this laudable goal by asking the addiction medicine community to embrace their novel disease model “Precision Behavioral Management (PBM) or “Precision Addiction Management (PAM), coupling genetic testing with Pro-Dopamine Regulation as a frontline gene guided therapy to provide early identification of RDS behaviors. We believe that to ignore the role of brain circuitry, especially as it is related to net dopamine release and or function at the reward center of the brain, will constitute scientific negligence. We believe that this Trieste, amongst others, is one more nail in the coffin of controlled drinking, for example, and the sin concept of all addictive behaviors removing stigma [145,146,147,148,149,150,151].

## Figures and Tables

**Table 1:** Brain Scan Tools.

Electrophysiology electric signals:
- Delayed p300 latency
- Decreased voltage of p300
- Abnormal polysomnography i.e., /increased nocturnal movement, decreased REM, decrease sleep efficiency
SPECT scans:
- Blood flow Single-photon emission computed tomography
- Decreased prefrontal lobe and temporal lobe circulation
- Decreased cerebral circulation
- EEG, delta wave, beta wave
fMRI:
Anatomy Functional magnetic resonance imaging
- Abnormal diffuser tensor imaging
- Abnormal fiber connections
- Abnormal neuropsychological tasks
- Hypo-activation of neuronal networks
- Prefrontal, frontal, parietal regions
MRI:
- Smaller brain volume in 5 subcortical areas, including amygdala, hippocampus, etc.
PET scans:
- Metabolism Positron emission tomography
Abnormal metabolism of dopamine and its transporters
- Abnormal binding to D2 receptors
MEG:
- Increased phase coherence and beta in gamma frequencies
